# Good practices for ultrasound examinations in gynecology and obstetrics during the COVID-19 pandemic: Number 1 - Janeiro 2021

**DOI:** 10.1055/s-0041-1723082

**Published:** 2021-01-29

**Authors:** Fernando Maia Peixoto Filho, Adriana Gualda Garrido, Anselmo Verlangieri Carmo, Eduardo Becker Júnior, Guilherme de Castro Rezende, Heron Werner Junior, Joffre Amin Junior, Jorge Roberto Di Tommaso Leão, Luciano Marcondes Machado Nardozza, Luiz Eduardo Machado, Manoel Alfredo Curvelo Sarno, Patricia El Beitune, Pedro Pires Ferreira Neto, Sergio Kobayashi, Fabricio da Silva Costa

**Affiliations:** 1 Universidade Federal de Uberlândia, Uberlândia, MG, Brazil; 2 Faculdade de Medicina, Universidade Federal de Brasília, Brasília, DF, Brazil; 3 Universidade Federal de Mato Grosso, Cuiabá, MT, Brazil; 4 Universidade Federal do Rio Grande do Sul, Porto Alegre, RS, Brazil; 5 Faculdade da Saúde e Ecologia Humana, Vespasiano, MG, Brazil; 6 Universidade do Rio de Janeiro, Rio de Janeiro, RJ, Brazil; 7 Universidade Federal do Rio de Janeiro, Rio de Janeiro, RJ, Brazil; 8 Universidade do Estado do Amazonas, Boca do Acre, AM, Brazil; 9 Universidade Federal de São Paulo, São Paulo, SP, Brazil; 10 Escola Bahiana de Medicina e Saúde Pública, Salvador, BA, Brazil; 11 Universidade Federal da Bahia, Salvador, BA, Brazil; 12 Universidade Federal de Ciências da Saúde de Porto Alegre, Porto Alegre, RS, Brazil; 13 Universidade de Pernambuco, Recife, PE, Brazil; 14 Faculdade de Medicina, Universidade de São Paulo, São Paulo, SP, Brazil; 15 Faculdade de Medicina, Universidade de São Paulo, Ribeirão Preto, SP, Brazil

## Key points

- Routine ultrasound examinations by specialists are an essential part of prenatal care and should be performed even in the extraordinary situation of the COVID-19 pandemic.
- The routine of obstetric and gynecological ultrasound services during COVID-19 pandemic period was changed to increase the protection of the patient, her fetus and her family, as well as of health professionals involved in care.
- The impossibility of maintaining the recommended distance during the ultrasound examination requires rigorous and systematic precautions, because patients and health professionals are exposed to COVID-19 infection.
- Given the risk of COVID-19 transmission, the performance of ultrasound examinations in gynecology and obstetrics must be based on a priority assessment and in accordance with Febrasgo protocols.
- COVID-19 infection within health services must be avoided at all costs. Part of this strategy involves the surveillance of employees in the sector and the systematic use of personal protective equipment (PPE).

## Recommendations

- Obstetric and gynecological ultrasound services must prevent the scheduling of appointments in person.
- Obstetric and gynecological ultrasound services should prepare informative material on infection prevention in the form of leaflets, use iconographic material in the various environments of the ultrasound sector and apply questionnaires about COVID-19 to patients to define the infection status.
- Patients must undergo screening before entering an ultrasound unit in order to check if they are already infected or have any suspicion for COVID-19.
- The ultrasound sector should provide information about specific training on hygiene, use of PPE and equipment handling for healthcare professionals, including doctors, nurses, technicians, cleaning, maintenance and administrative staff, among others.
- Symptomatic patients not severely immunosuppressed: reschedule after ten days from the onset of symptoms.
- Patients living with people with symptoms of COVID-19: reschedule after 14 days.
- Positive polymerase chain reaction (PCR) patients who remain asymptomatic, not severely immunosuppressed: exempt from the scheduled appointment and reschedule ten days after the first positive real-time PCR (RT-PCR) test for COVID-19.
- Patients with positive PCR who remain asymptomatic and severely immunosuppressed: exempt from the scheduled appointment and reschedule 20 days after the first positive RT-PCR test for COVID-19.
- Aiming to reduce the time of direct contact with the patient, make videos of anatomical regions or capture specific screenshots and take biometric measurements offline. An experienced ultrasonographer may be chosen to perform the exams, thereby reducing the exam time and the need for a second opinion.

## Background

The COVID-19 pandemic period changed the routine of obstetric and gynecological ultrasound services, 1 as well as the routine in all other women's health care services. 2 3 Such changes involve a series of protective measures that will be highlighted in this regulation.

Based on the care, safety and prevention of COVID-19, procedures to improve the protection of the patient, her fetus and her family and of health professionals involved in the care are mentioned. Routine ultrasound examinations by specialists are an essential part of prenatal care and should be performed even in the extraordinary situation of the COVID-19 pandemic. Precautions must be rigorous and systematic in the practice of this clinical activity, mainly because there is exposure of the patient and health professionals, given the impossibility of maintaining the recommended distance during the procedure. 1

The information found in this document is based on the best practices recommended by National and International Diagnostic Imaging Societies and guided by two main axes:

1. Prevention against the expansion of COVID-19 during the performance of ultrasound examinations of women;
2. Protection of the patient and health care professionals in the ultrasound sector in order to maintain a safe workflow from the scheduling to the performance of exams. Note that conducts provided here can be adapted depending on the availability of resources and infrastructure.

## Routine of obstetric and gynecological ultrasound exams in the context of the COVID-19 pandemic

### How should the scheduling of exams in gynecology and obstetrics be during the COVID-19 pandemic?

The guidelines to avoid scheduling in person are as follows: 4 5 6

- Exams must be scheduled by phone or the internet;
- The professional of the service unit, previously guided by doctors, should contact the patient on the eve of the exam to check about symptoms and risk factors for COVID-19 ( Chart 1 );
- Scheduling times should have a safe interval in between to avoid the crowding of patients in the waiting room;
- If patients have already done tests before, they should be instructed to bring them;
- If patients spontaneously appear at the medical ultrasound unit without prior appointment, they must return with a rescheduling and be informed that their prenatal care will not be affected;
- In cases that a rescheduling is impossible, when these patients present some type of symptom, possibly related to a respiratory infection, such as cough, fever or sore throat, they must undergo screening immediately for guidance to the best solution.

**Chart 1.** Guidelines for screening patients for coronavirus infection in elective exams.

“Before service, you must answer some questions. For everyone's protection, it is important that you answer honestly”. It is important to ask one question at a time and wait for each answer.
• Do you have the flu? • Are you coughing? • Do you have a runny nose? • Do you have a sore throat? • Do you feel body ache? • Can you smell food and other things? • Can you taste food? • Do you have a fever? • Do you have chills? • Do you have the flu? • Do you feel shortness of breath? • Do you have diarrhea? • Have you had contact with someone who tested positive for COVID-19 in the past 14 days? • Have you had contact with someone who has been hospitalized for flu or pneumonia in the past 14 days?

Source: Adapted from the Ministry of Health (2020). 7

### In the screening of patients for elective exams, what is the procedure to avoid COVID-19 transmission?

Before entering an ultrasound unit to do their exams, patients must undergo screening in order to check if they are already infected or have any suspicion for COVID-19.

This screening should be done with all preventive care:

- Mandatory use of masks;
- Control of the entry, exit and transit of patients;
- Reservation of a specific place in the waiting room;
- Access to information on respiratory etiquette.

Informative material on the prevention of infection should be prepared in the form of leaflets, iconographic material should be used in the various environments of the ultrasound sector and questionnaires about COVID-19 should be applied to patients to define the infection status.

Patients should be investigated for symptoms related to the risk of COVID-19 ( Chart 1 ). 7 At this time, their temperature should be measured. In case of cough, high fever or shortness of breath, these patients must undergo specific screening for COVID-19 and evaluation by a health professional in accordance with local protocols. 6

### What are the recommendations for screening patients with possible coronavirus infection in elective exams?

- Symptomatic patients not severely immunosuppressed: reschedule after ten days from the onset of symptoms. 8
- Patients living with people with symptoms of COVID-19: reschedule after 14 days. 8
- Patients with positive PCR who remain asymptomatic and not severely immunosuppressed: exempt from the scheduled appointment and reschedule ten days after the first positive RT-PCR test for COVID-19. 8
- Patients with positive PCR who remain asymptomatic and severely immunosuppressed: exempt from the scheduled appointment and reschedule 20 days after the first positive RT-PCR test for COVID-19. 8

**In such cases, follow-up can be done by direct communication with the patient or preferably by remote technology means.**

### How to assess the priority of ultrasound exams in the context of the COVID-19 pandemic?

Exams must be analyzed using three types of priority: 6

- Exams that must be done immediately without delay;
- Exams that can be postponed without harm to the patient's care;
- Exams that can be suspended during the pandemic period.

From this choice of priorities, the following points must also be considered:

- The routine of ultrasound examinations covering specific periods of pregnancy in the course of the first or second semester; 9 10
- Any history of genetic or structural abnormalities, 9 placental insufficiency (preeclampsia, fetal growth restriction), 11 12 previous preterm birth, twin pregnancy, 13 as well as any previous maternal diseases harmful to pregnancy; 14
- If there are any risk factors that arose during the pregnancy period.

In these cases, the recommendations for performing obstetric and gynecological ultrasounds are as follows:

- Select an experienced ultrasonographer to perform these exams, thereby minimizing the need for a second opinion;
- Follow Febrasgo protocols to determine the time of exams in the first and second trimester of pregnancy and for the performance of gynecological exams;
- If possible, for reduced time of direct contact with the patient, make videos of anatomical regions or capture specific screenshots and take biometric measurements offline.

### What is the routine of obstetric exams for patients without preexisting maternal or fetal comorbidities?

- In the first trimester (preferably between 11 and 14 weeks), the following should be evaluated: location of pregnancy, viability, number of fetuses and chorionicity, date of pregnancy, fetal anatomy and screening for aneuploidy and preeclampsia. 6
- Perform morphological ultrasound at week 20-22 for the assessment of fetal static, cardiac activity, biometrics, basic fetal anatomy, fetal wellbeing and placental location. 6
- Perform fetal growth screening exams during the third trimester. 6

Patients with comorbidities should use the guidelines provided in the previous item. However, the monitoring must be more rigorous and follow the specific protocols for each condition. 6 15

### How to proceed with ultrasound exams in hospitalized patients with suspected or confirmed cases of COVID-19 infection?

The ultrasound examination must be done by an experienced doctor, preferably at the bedside. The following must be evaluated: fetal statics, cardiac activity, biometrics, amniotic fluid volume, placenta and umbilical artery doppler velocimetry. Another important point is the assessment of fetal growth within four weeks after recovery.

### How should the waiting environment for exams be organized in patients with and without suspected/confirmed COVID-19?

#### Ultrasound sector waiting room

In the waiting room environment, the distance of two meters between seats must be preserved with a maximum of one person in the examination room. Children or other relatives should not be allowed in this area. Patients should be advised to go unaccompanied to the examination site. To reduce the risk of transmission among health professionals, it is recommended that people who fit into a risk group do not stay in the waiting room, which is for exclusive use of scheduled patients.

#### Creation of an isolation room

The isolation room is recommended for patients with suspected COVID-19 or confirmed cases who need to perform a gynecological or obstetric examination in the ultrasound sector. These patients should be examined in an exclusive room with a bathroom. 3 It is important to ensure thorough cleaning of the equipment used.

#### Cleaning of the ultrasound room and its devices 6

Cleaning with appropriate disinfectant should be performed every morning in the morning, including the ultrasound monitor, computer keyboard and mouse, stretcher rails, transducer support, gel container, door handles, buttons, light switches, chairs and countertops.

Reduce the number of accessories on site that are not being used. These surplus accessories must be removed and kept in a clean, closed place. As for transducers connected to the ultrasound, only one trans-abdominal and one transvaginal are sufficient, used as required.

In the case of patients with suspected/probable/confirmed infection, the equipment should preferably be separated and used exclusively. It should always be sanitized, preferably with single-use gel packets, avoiding the usual containers. Prefer the use of disposable gloves and equipment.

### What are the precautions with equipment and how should PPE be used in the ultrasound sector?

Some care is essential for professionals who have direct contact with patients or even with potentially infectious materials. This directly involves the use and access to PPE: 5

- Pay attention to the time of use and removal of PPE; with gloves, for example, in order to avoid self-contamination and that of third parties, as the chance of contamination is greater if removal of this equipment is inappropriate;
- Discard and perform the proper maintenance of non-disposable PPE.

Likewise, information on specific training on hygiene and instrument handling for health professionals should be provided, including doctors, nurses, technicians, cleaning, maintenance and administrative staff, among others.

Some important guidelines: 15

- Make sure the ultrasound professional is experienced and has undergone infection control training;
- Pay strict attention to hand hygiene, before and after contact with the patient. If you cannot wash your hands, use a disinfectant or an appropriate sanitizing product;
- Give preference to single-use gel packets instead of using the gel directly from containers;
- Use the transducer cover for non-endoluminal probes;
- Always aim at reducing the exam duration.

Use of more specific equipment to treat patients with COVID-19 -confirmed or suspected/symptomatic/asymptomatic: 5 6 15

- Mask:
  - The patient must wear a face mask to avoid and/or reduce transmission by droplets;
  - The professional must follow the same hygiene rules and mandatorily use the facial mask;
  - Remove the mask carefully and always proceed with hand hygiene.
    - Wash your hands with water and soap/ detergent for at least 20 seconds;
    - Use specific products with alcohol in the concentration of 60%-95%;
    - The ultrasound operator should preferably use a three-layer surgical mask.
- Eye protection
  For eye protection, the use of a disposable facial protector that can protect the front and sides of the face is recommended. It should always be used when professionals are in the patient's room and in the service area.
  Two important aspects to be highlighted:
  - Glasses and contact lenses for personal use are not considered an appropriate eye protection;
  - Eye protection must always be removed when leaving the service area.
- Gloves
  Appropriate care with gloves must include the following:
  - Always be clean when entering the service room;
  - Be removed and discarded when leaving the service area;
  - Hands should be cleaned after removing the gloves;
- During the ultrasound examination, disposable gloves, preferably latex-free, should be used, which will be changed after the care of each patient. 15
- Aprons
  Disposable and non-disposable gowns can be used in patient care areas. Disposable gowns that can be placed in an appropriate disposal container before leaving the service area should be preferred.

### Environmental infection control

It is mandatory to follow procedures for cleaning and disinfecting the ultrasound environment consistently and correctly. The control of environmental infection must aim at reusable materials that will be disinfected according to instructions provided by the manufacturer and supported by its installation and use policies. This also includes the care and management of objects and clothing of the team of professionals. Unusable material must be discarded according to health surveillance rules.

### How to deal with employees and collaborators of the health team with COVID-19?

In case of confirmed cases of COVID-19 infection in the hospital service unit, recommendations are the following:

- Put part of the health team to work remotely in order to avoid the simultaneous contamination of a relevant part of professionals and thereby, not impact the maintenance of services;
- For professionals who already show symptoms of COVID-19 infection, flexible medical leave policies consistent with public health guidelines should be implemented.

## Final considerations

This regulation covers different procedures that must be followed in the pandemic period of COVID-19 for the performance of obstetric and gynecological ultra-sounds in order to monitor and preserve the health of pregnant women and their fetuses, and of all professionals involved.

National Specialty Commission for Ultrasonography in GO of the Brazilian Federation of Gynecology and Obstetrics Associations (FEBRASGO)

President:

Fabricio da Silva Costa

Vice-President:

Eduardo Becker Júnior

Secretary:

Heron Werner Júnior

Members:

Adriana Gualda Garrido

Anselmo Verlangieri Carmo

Fernando Maia Peixoto Filho

Guilherme de Castro Rezende

Joffre Amim Junior

Jorge Roberto Di Tommaso Leão

Luciano Marcondes Machado Nardozza

Luiz Eduardo Machado

Manoel Alfredo Curvelo Sarno

Patricia El Beitune

Pedro Pires Ferreira Neto

Sergio Kobayashi
